# Delivering Perinatal Health Information via a Voice Interactive App (SMILE): Mixed Methods Feasibility Study

**DOI:** 10.2196/18240

**Published:** 2021-03-01

**Authors:** Lisa Militello, Emre Sezgin, Yungui Huang, Simon Lin

**Affiliations:** 1 Martha S Pitzer Center for Women, Children & Youth College of Nursing The Ohio State University Columbus, OH United States; 2 Research Information Solutions and Innovation The Abigail Wexner Research Institute Nationwide Children's Hospital Columbus, OH United States

**Keywords:** perinatal care, infant mortality, health education, mobile health, feasibility studies, family, mobile phone, webcasts as topic, user-computer interface

## Abstract

**Background:**

Perinatal health care is critically important for maternal health outcomes in infants. The United States fares considerably worse than comparable countries for maternal and infant mortality rates. As such, alternative models of care or engagement are warranted. Ubiquitous digital devices and increased use of digital health tools have the potential to extend the reach to women and infants in their everyday lives and make a positive impact on their health outcomes. As voice technology becomes more mainstream, research is prudent to establish evidence-based practice on how to best leverage voice technology to promote maternal-infant health.

**Objective:**

The aim of this study is to assess the feasibility of using voice technology to support perinatal health and infant care practices.

**Methods:**

Perinatal women were recruited from a large Midwest Children’s Hospital via hospital email announcements and word of mouth. Owing to the technical aspects of the intervention, participants were required to speak English and use an iPhone. Demographics, patterns of technology use, and technology use specific to perinatal health or self-care practices were assessed at baseline. Next, participants were onboarded and asked to use the intervention, Self-Management Intervention–Life Essentials (SMILE), over the course of 2 weeks. SMILE provided users with perinatal health content delivered through mini podcasts (ranging from 3 to 8 minutes in duration). After each podcast, SMILE prompted users to provide immediate verbal feedback to the content. An exit interview was conducted with participants to gather feedback on the intervention and further explore participants’ perceptions of voice technology as a means to support perinatal health in the future.

**Results:**

In total, 19 pregnant women (17 to 36 weeks pregnant) were consented. Themes identified as important for perinatal health information include establishing routines, expected norms, and realistic expectations and providing key takeaways. Themes identified as important for voice interaction include customization and user preferences, privacy, family and friends, and context and convenience. Qualitative analysis suggested that perinatal health promotion content delivered by voice should be accurate and succinctly delivered and highlight key takeaways. Perinatal health interventions that use voice should provide users with the ability to customize the intervention but also provide opportunities to engage family members, particularly spouses. As a number of women multitasked while the intervention was being deployed, future interventions should leverage the convenience of voice technology while also balancing the influence of user context (eg, timing or ability to listen or talk versus nonvoice interaction with the system).

**Conclusions:**

Our findings demonstrate the short-term feasibility of disseminating evidence-based perinatal support via podcasts and curate voice-captured data from perinatal women. However, key areas of improvement have been identified specifically for perinatal interventions leveraging voice technology. Findings contribute to future content, design, and delivery considerations of perinatal digital health interventions.

## Introduction

### Background

Nearly 60% of maternal deaths are preventable [1], and infant mortality rates are approximately 71% higher in the United States than in other comparable countries [2]. Race/ethnicity, low income, and chronic stress are associated with pregnancy-related complications and poor maternal and infant mortality statistics [3-6]. To set the stage for long-term health and well-being of the mother and the infant [7-9], the American College of Obstetricians and Gynecologists (ACOG) and the American Academy of Pediatrics emphasize the importance of maternal perinatal care and infant preventive care. The benefits of quality perinatal health care are well established, reducing the risk of pregnancy complications, rates of low birth weight infants, and infant mortality rates [5,10,11]. However, the United States fares much worse in preventing pregnancy-related complications than most other developed countries, despite spending more than any other country on hospital-based maternity care [12,13]. Growing concerns regarding maternal-infant health outcomes, patient satisfaction, access to quality prenatal care, and costs have increased interest in alternative models of prenatal care [14]. Given the broad and ubiquitous nature of technology, digital health tools have the potential to advance perinatal care and empower women to engage in the provision of care while maintaining expert recommended standards of care [15,16].

Evidence shows that pregnant women and those with young children are accustomed to readily available information using digital technologies and desire better access to information offered by health professionals [17]. Earlier efforts to supplement perinatal care with digital health tools have demonstrated variable levels of technological complexity. One of the most notable public health campaigns for perinatal health is Text4baby. With more than 250 messages tailored to pregnant women and new mothers, Text4baby represents one of the first empirically supported mobile health campaigns to reach over 685,000 mothers through text messaging [18]. Similarly designed for national scalability, Expect With Me follows the same schedule as individual prenatal care from week 14 of pregnancy and follows ACOG recommendations for clinical practice implemented through group prenatal care supplemented with information technology [15,19]. The obstetric OB Nest program proposes a reduced number of prenatal visits (ie, 8 onsite obstetric appointments; 6 virtual nurse visits) for low-risk pregnant women by leveraging technology (eg, fetal heartbeat and blood pressure home monitoring devices; web-based social support) to demedicalize the pregnancy experience and provide care within patients’ daily lives [20,21]. Similar efforts to reach women in their daily lives, researchers found it feasible to use an embodied conversational agent (ie, animated conversational character simulating face-to-face interaction) accessed over the web, *Gabby*, to promote preconception health, healthy eating, and stress management [22,23]. Collectively, a review of perinatal care and telemedicine/eHealth suggests that digital tools may be beneficial in empowering patients and promoting value-based health care, yet ongoing efforts are needed to provide evidence specific to health outcomes, satisfaction, and cost and reflect a constantly evolving digital landscape [24]. As a digital health intervention tool, voice technology has recently been explored as a modality for delivering information to support health and well-being [25]. For the purposes of this paper, we define voice technologies as digital tools and devices that enable bidirectional communication of information through speech (eg, conversational agents, dialog systems using audio content or text-to-speech over smart speakers, smartphone voice assistants, voice-based apps). Voice technology interventions that rely on listening and speaking interactions differ from visual intervention predecessors and warrant further research to understand how users interact and consume information [26].

### Aims of This Study

The primary aim of this study is to assess the feasibility of delivering perinatal health education via voice among a group of perinatal women. To explore the potential of voice technology in maternal-infant health, we aimed to assess the feasibility of a voice technology mobile app prototype, Self-Management Intervention–Life Essentials (SMILE), among a group of perinatal women. Following expert recommendations [27], we defined feasibility through 4 general domains: (1) acceptability, (2) demand, (3) practicality, and (4) adaptation. We examined the ability of the application to retrieve and deliver perinatal health information through spoken words (eg, podcasts) and prompt and audio-capture participant reactions to intervention content immediately following the podcasts. Before efficacy testing, we sought to understand participants’ tolerability of the platform, appropriateness and interest in the content and delivery, and how participants used the system.

### Intervention: SMILE

As an initial prototype, SMILE was created using the input from the literature. From pregnancy through a baby’s first birthday, the literature collectively identifies the following categories necessary in perinatal education: information regarding infant/babies’ needs, postpartum care and postpartum depression, baby’s feeding/breastfeeding, strategies to manage the couple’s relationship, mobile/digital resources with links to reliable documents, and a list of useful contacts/professional resources [28,29]. In addition to traditional resources such as family and close friends, new parents use alternative contemporary channels to find perinatal (pregnancy/parenting) information to include mobile and internet-based resources [30]. Specifically, digital health interventions have demonstrated the ability to provide women with perinatal support when they most need it (ie, immediate) and/or when they have opportunities to access content (ie, support is more readily accessible than clinic visits alone) [17,28].

Podcasts are increasingly being used for education, both to providers and patients, with demonstrated feasibility, acceptability, and reach [31,32]. We leveraged the convenience of podcasts to deliver SMILE content. SMILE was developed to retrieve content from the long-standing, evidence-based Dr Mike PediaCast program affiliated with the clinical setting for the study [33]. PediaCast is a parent-facing podcast that provides relevant information and news to parents by answering listener questions, interviewing pediatric experts, offering overviews of research, and providing the latest news on pediatric health–related topics.

**Figure 1:**
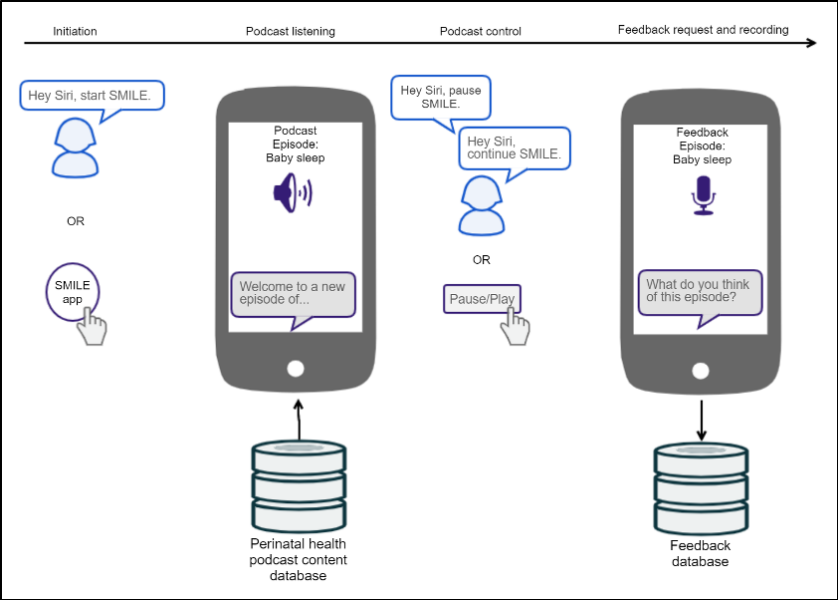
Self-Management Intervention–Life Essentials interactivity highlights.

For initial testing and because of time and monetary constraints, SMILE leveraged existing voice technology services (ie, Siri, podcasts) and was designed as a mobile app for use on the iPhone platform. SMILE could be initiated through voice on the user’s phone (eg, “Hey Siri, start SMILE”) or by launching the app through touch/tap, either method would then start a preselected podcast. Intervention podcast topics were selected based on their relevance to infant care practices, prenatal care practices related to improved infant outcomes, and the duration of podcasts. Although evidence suggests options for users to tailor/personalize the intervention, podcasts were delivered sequentially to gauge user perceptions of various topics and durations. Users were able to listen to podcast in the background (ie, screen off), with an option to play/pause with voice command through Siri (“start SMILE” to initiate the app and it automatically starts the podcast, “Pause SMILE” to pause episode, and “Continue SMILE” to play where left off). Finally, upon completing each podcast episode, the app (through spoken language) asked users to provide feedback by answering (verbally) 2 brief questions. User feedback was collected by the app through voice recordings. Figure 1 highlights SMILE functionalities and how participants could use the app during the study.

### Theory

The underlying tenets of the proposed innovation are theoretically grounded in cognitive load theory [34] and the technology acceptance model (TAM) [35]. Cognitive load theory asserts that when experience overloads working memory capacity, learning is impaired [34]. Learning is better supported when content is broken down into smaller, more manageable pieces. As such, SMILE podcasts ranged from 3- to 8-minute chunks, each episode slightly longer, but allowed users the option to stop after one episode or continue listening. Qualitative aspects of the study were guided by the TAM (discussed in detail in the *Data Collection* section).

## Methods

### Design

We conducted a 2-week within-subject feasibility study with 19 perinatal women.

### Recruitment, Sample, and Setting

Recruitment occurred over a 1-month period (April to May 2019) at the Nationwide Children’s Hospital, Columbus, Ohio. Participants were invited to participate through hospital email announcements and word of mouth. Interested persons were screened before enrollment. To advance the health of women and infants, a two-generation approach acknowledging the interrelated health between mother and infant is critical. Therefore, the main study inclusion criterion was perinatal women ≥18 years of age, either pregnant or having an infant less than 1 year of age. Owing to technical aspects of the intervention, participants were also required to be English speaking and an iPhone user. In appreciation for participant time and feedback, compensation was provided for baseline survey completion and downloading the app (US $10), field testing the app, and participating in an exit interview (US $20) for a possible US $30 total.

### Data Collection

Upon written informed consent, participants completed baseline surveys that captured demographics, patterns of technology use, and technology use specific to perinatal health and/or self-care practices (Multimedia Appendix 1). Participants were then onboarded and instructed to download and use the app over a 2-week period. To prevent nonrecruited users from downloading and using the app, participants were provided with a link to download SMILE along with an assigned entry code necessary to launch the app. To broadly assess app usage, data were collected from app store analytics (ie, number of active devices, number of impressions). Participants’ individual app use (eg, demand) was also collected (ie, podcast number, duration of app used) to understand how participants progressed through the intervention. Qualitative data reflective of participants’ acceptability, perceptions, and attitudes toward the intervention were obtained through 2 channels. First, immediately following each podcast, participants were asked 2 questions and responses were audio-captured and recorded through the app. Second, participants were invited to participate in a semistructured exit interview following the 2-week intervention field test. Blending formal structured and unstructured interviewing techniques, semistructured techniques are widely used in formative research studies that provide researchers with information on the acceptability of intervention components [36]. The exit interview was informed by the TAM [35] and a scale to assess burden [37] to gather feedback and attitudes toward the technology (Multimedia Appendix 2). Although scheduled and drop-in sessions were offered for exit interviews within the hospital setting, 25% (3/12) of participants completed face-to-face interviews, whereas the rest opted to complete interviews via audio/videoconferencing. Qualitative feedback was audio recorded, deidentified, transcribed verbatim, and verified against actual recordings by study staff. Field notes taken during the interviews were used to supplement the transcripts.

### Data Analysis

Quantitative data were analyzed using descriptive statistics. Two researchers performed thematic analysis of qualitative data [38,39]. Owing to the study design, 2 forms of qualitative data were evaluated. First, qualitative data collected immediately following each podcast episode queried participants about the intervention content. Second, qualitative data collected via exit interviews provided participant feedback on the overall intervention. For all qualitative data, researchers first became familiar with the qualitative data, which involved multiple readings of transcripts, but did not code any data. Third, key concepts were identified. Color-coding strategies, both manually and with Excel spreadsheets, were employed to highlight various concepts and generate initial codes. Coded data were reviewed by research team members who compared and contrasted their independent findings. The initial codes were iteratively modified in the process of open coding to capture information relevant to the research question. Identified thematic findings were reviewed and modified within the context of the larger data set to ensure that the themes were cohesive, yet distinct [39]. Final, prominent themes were discussed between the 2 authors until a consensus was reached. To gauge user perceptions of episode content and delivery, an additional sentiment analysis was independently performed for app-collected feedback. Participant feedback responses were coded −1 for negative, 0 for neutral, and 1 for positive. Disagreements were resolved through discussion and consensus.

### Ethical Consideration

Ethics approval was obtained from the participating hospital, Nationwide Children’s Hospital Internal Review Board (IRB #00000159). Participation was strictly voluntary, and participants were informed of their right to withdraw at any time without penalty. Participant data were deidentified and stored on a secure server.

## Results

### Demographics

Collectively, 19 participants (17 to 36 weeks pregnant) were consented and completed baseline surveys, 18 downloaded the SMILE app, 17 used the app, and 12 participated in the exit interviews. The sample was predominantly White (15/19, 79%), married (19/19, 100%), between 25 and 34 years of age (16/19, 84%), and pregnant with their first child (12/19, 63%). Although education level and occupation were not formally assessed, some of the participants self-identified as nurses or social workers or in *admin* during the exit interviews.

At baseline, the top 3 resources participants used for pregnancy-related information included calling their health care provider (7/30, 23%), searching the web (7/30, 23%), or using smartphone apps (6/30, 20%). The top 2 most cited apps used to support perinatal health were Ovia (6/42, 14%) and What to Expect (5/42, 12%). Resources least used included an information packet provided by a health care provider (2/30, 7%), calling a friend (2/30, 7%), or using a doula (1/30, 3%). Calendar was the most cited (6/14, 43%) app used to support everyday life. More than half of our participants reported using voice assistants before this study (11/19, 58%). Participant-reported voice technology data are highlighted in Table 1.

**Table 1:** Participant-reported voice technology engagement (baseline).

Category				Value, n (%)
**Smartphone-specific voice technology engagement**				
	**Duration of voice use (n=11 participants)**			
		1-3 months	2 (18)	
		3-12 months	1 (9)	
		1-3 years	6 (55)	
		>3 years	2 (18)	
	**Top 3 interactions (n=21 responses)^a^**			
		Weather	4 (14)	
		Phone calls	3 (10)	
		Timer	3 (10)	
		Date/time	2 (7)	
		Information seeking	2 (7)	
		Texting	2 (7)	
		Music	2 (7)	
		Reminders	2 (7)	
		Map	1 (5)	
**Smart speaker–enabled or voice-enabled technology engagement**				
	**Duration of voice use (n=10 participants)**			
		1-3 months	2 (20)	
		3-12 months	2 (20)	
		1-3 years	5 (50)	
		More than 3 years	1 (10)	
	**Top 3 interactions (n=26 responses)^a^**			
		Weather	6 (23)	
		Playing music	6 (23)	
		Smart house (lights, switches, and thermostat)	5 (19)	
		Timer	4 (15)	
		Information seeking	2 (8)	
		Cooking recipes	1 (4)	
		Conversion or calculation	1 (4)	
		Smart television access (search movie)	1 (4)	

^a^Participants offered more than one response.

### App Usage

Unfortunately, the system failed to capture data related to how the app was initiated (touch/tap or voice), time of day when the app was being used, or app usage duration. Therefore, app usage was determined from participant feedback captured immediately after each podcast episode listened to by the participants. Using these data, SMILE was able to deploy 239 podcasts across 17 participants in a 2-week timeframe (Table 2).

**Table 2:** Sentiment ratings for podcast episodes grouped by category.

Category	Proportion of podcast episodes available by category^a^ (n=23), n (%)	Proportion of listens by category^a^ (n=239), n (%)	Frequency of response for question 1 (“What did you think of this content?”)	Content positively rated (question 1), n (%)	Frequency of response for question 2 (Interest in hearing more)	Interest in hearing more (question 2), n (%)
All	23 (100)	239 (100.0)	237	205 (86)	238	209 (86)
Sleep	8 (35)	81 (33.9)	81	71 (88)	80	67 (84)
Pregnancy	7 (30)	72 (30.1)	71	59 (83)	72	66 (92)
Parenting	7 (30)	72 (30.1)	75	71 (95)	74	60 (81)
Breastfeeding	4 (17)	36 (15.1)	35	32 (91)	36	33 (92)

^a^Some percentages will sum to >100%, as some categories overlap.

Participants were required to listen to the podcasts in order. The response rate to the 2 questions prompted after each podcast was 60.1% (475/782). The least amount of words voice captured from participant feedback was 2 (17 characters), while the most amount of words voice captured from participant feedback was 134 (725 characters). Participants most often used 18 words (12/239, 5.0%) in voice feedback, with a median of 34 words per response (IQR 37.3, min-max 20-57.3). Participant feedback was coded positive (1, *good*, *helpful*), neutral (0, *not sure*), or negative (−1, *not relevant*, *not helpful*). The full podcast episode list and ratings are given in Multimedia Appendix 3. From the 475 responses collected by the system, 87.2% (414/475) comments were positive. The average sentiment rating for question 1 was favorable at 86.8%, and the average interest in hearing more was positive (85.8%). Despite the positive feedback, the number of users per episode was reduced by nearly half during episode 15. Each category was covered by at least two podcasts during the first 15 episodes. Therefore, the notable attrition does not necessarily reflect loss of interest in a category but may reflect decreased use of the intervention over time. Furthermore, it is difficult to say with certainty that missing data (eg, no user feedback for an episode) were truly indicative of missing data or technical glitches. From the app-captured participant feedback, at least three instances were noted where the technology failed to capture participants’ initial responses:

I already answered questions for this episode, and it didn’t save.

Participant 5, episode 14

### Podcast Qualitative Feedback

Participant feedback captured by voice recordings following each podcast was analyzed qualitatively. Sentiment was gauged from 0% (poor/negative) to 100% (good/positive). Themes identified from content feedback include (1) establishing/transitioning routines, (2) expected norms and tempered expectations, and (3) key takeaways. The themes and representative quotes are given in Table 3.

**Table 3:** Examples of qualitative feedback to podcast episodes by theme.

Theme/subtheme	Example (feedback on podcast)	Interested in hearing more
Routines	“Like it’s very relevant to a new mom because I currently have a 16-month-old and pregnant and I did not know how difficult getting a sleep routine down was. So, I think hearing I didn’t know that routine made such a difference and once I started routine. It was very helpful.” (Participant 17, episode 8)	“How and when to start sleep routines with younger babies” (Participant 8, episode 9)
Expected norms/realistic expectations	“I’ve had a lot of friends tell me that it’s good that I’m considering breastfeeding, but not to feel so ashamed if it doesn’t happen the way that I want it to go and it’s okay.” (Participant 15, episode 14)	“I think it might be helpful to hear more about what to look for, how to treat it, when to call the doctor.” (Participant 17, episode 1)
Troubleshooting (subtheme)	“I like this episode a lot because he went through several different reasons for why the baby was crying before sleep and kind of troubleshooting to figure out what the issue was. It was nice to hear him describe several options that could’ve been the case. Yeah, I liked it a lot. I thought it was useful.” (Participant 10, episode 8)	“... what are some techniques to help soothe them rather than feed them and what are some cues as a mom to figure out that they really need fed or they just want attention.” (Participant 17, episode 19)
Provide objective, key takeaways	“I thought this episode was pretty detailed and gave good information. I would have liked to also hear what to look for when looking for signs of suffocation and then maybe have him defined supervision a little bit MORE ... very helpful to have an acronym to remember safe practices.” (Participant 15, episode 6)	“As I mentioned previously it would be helpful for parents to have more information about specific examples.” (Participant 11, episode 15)

#### Theme 1: Establishing/Transitioning Routines

Content about establishing routines and transitioning to having routines were of interest to the participants. For example, the ninth podcast, *Infant Sleep Problems*, was the fourth time that typical infant sleep patterns were discussed. However, participants’ feedback indicated that they would be interested in hearing more about the timing and strategies to establish sleep routines. Collectively, participants were interested in learning *when* it was safe to establish or transition their baby and strategies on *how* to go about it.

#### Theme 2: Expected Norms and Tempered Expectations

Participants were eager to hear information that validated their prior knowledge and experiences to gain *peace of mind*. Participants favored information that showed the pros and cons of various parenting techniques and infant care strategies. Anecdotal experiences can be perceived as positive if supported by accurate medical data:

More real-life examples of breast feeding and issues that go along with it.

Participant 6, episode 14

When caring for their newborn, participants wanted information about expected behaviors and strategies to determine infant needs, specifically infant *cues*. Feedback indicated that participants desired to learn more about alternative solutions to common parenting challenges.

#### Theme 3: Provide Key Takeaways

Participants favored podcasts that provided objective data to dispel myths. Often, information reinforced with statistics, data, or research has been favorably received. However, this information must be tempered by presenting the information in terms of the user’s understanding:

...it had a lot of good data, but it was almost too data heavy to stay focused on

what it was saying because it was statistic after statistic after statistic.

Participant 8, episode 2

Information tended to be favorably received if it provided succinct tips and was not *all over the place* or *lots of information* as in too general or too much.

### Exit Interview Findings

Exit interviews were focused on participant feedback and attitudes toward voice technology. Exit interview qualitative data were analyzed separately from app feedback qualitative data. Themes that emerged from coded exit interviews included (1) customization of user preferences, (2) privacy concerns, (3) family and friends, and (4) convenience and context.

#### Theme 4: Customize and User Preferences

Participants expressed their preferences for seeking information and learning. In exit interviews, participants spoke to the benefits of having information presented in more than one way, with at least three women self-identifying as visual learners:

If it’s something in depth you know, and I want to be able to... I am a visual learner to see the chart or the you know to comprehend it. I need both.

Participant 3

When I was looking at My Chart (patient portal), there have been times that I didn’t even remember what the test was called that they did. So, it would have been hard to recall that and then go through all of those tests... I like to see things versus hearing. I like a little bit of both, but I lean towards more visual so I like to be able to click through.

Participant 7

Prior experience and user familiarity with technology have emerged as relevant. Some described themselves as *not very tech savvy* and preferred more traditional methods such *writing things down*:

I prefer reading because it can, um, allow me to go at my own pace and I don’t miss information. Um, and then I can use that as notes. Like, I can take a snapshot of it, uh, and use that to reference back upon because when you hear stuff, it doesn’t necessarily, like you might not hear it the right way. Or, um, you know, you can’t remember exactly what was said.

Participant 2

Finally, participants advocated for expanding content to allow for more customized material and tailoring options. Thus, the creation of user profiles may be beneficial:

... being able to like choose their own content, um, not necessarily have to listen to the same thing multiple times in a row and same in different ways, you know? If there’s more options, then people can pick their own thing. I think that that will have more benefit and possible interaction with voice commands and that kind of stuff.

Participant 1

How, in my mind, I’m thinking when you go into Netflix, and you get a profile, and you pick, you know, my husband has a profile, I have a profile, and when you log onto that, they recommend certain shows to watch based off your past history. So, I guess if there’s a way that you could log into your Alexa as far as like your portal or your profile, and then it kind of like tracks your trends of things you ask or interests like music that you play.

Participant 8

#### Theme 5: Privacy

Privacy was brought up in an array of contexts. Privacy of voice technology was discussed within the context of information control and information sharing.

One participant stated this about digital privacy:

I don’t think I have a lot to hide... I’m not nervous about it. But I also feel like I probably should be a little bit more aware of it. Cuz I feel like on my phone it’s like you can be talking about something and then you get on your Facebook page.

Participant 2

Privacy was also mentioned with regard to context of voice:

I’m rarely in a quiet and alone place where I really can speak, to give a command to a phone, even if it were to recognize me more sometimes either maybe a privacy thing, or I don’t want everybody around me to know and I’m I just don’t want to be broadcasting is what I’m learning about.

Participant 11

#### Theme 6: Family and Friends

Exit interviews captured voice-enabled devices used in the home setting (eg, smart speakers, smart switches, smart thermometers) other than smartphones alone. Voice technology use was perceived differently depending on the family member using it.

Several participants spoke of the influence their spouses, particularly their husbands, had on technology uptake. In most cases, spouses promoted the uptake of technology; however, they could also pose as a barrier.

Responses suggested that husbands were the drivers of technology within the household:

Yes, absolutely. Yeah. If he wasn’t super techno savvy, then I probably honestly wouldn’t have an Alexa and I wouldn’t have done the smart lights or things like that. He’s definitely the driver behind because he likes being the new and cutting edge and trying it out and that type of stuff.

Participant 9

Yeah we don’t use Alexa at home. Cuz mostly my husband is a security person, he doesn’t like it. I’d use it myself a lot more if it were not for him.

Participant 10

Husbands could also be engaged in the perinatal process through voice:

I could absolutely see my husband going like Alexa, tell me about my baby today and she’s rattling off like your baby should be about this weight and should be eating this much and all this like he would totally absolutely use it. I would probably use it as well if the information is valuable.

Participant 9

Participants voiced similar concerns about their children’s safety, as infants/siblings learn to speak and role model others, exposure to inappropriate material, and becoming addicted to technology. Others have suggested a need for parental control on voice-enabled devices:

I’d rather the kids wait until they’re older to use it, especially unsupervised. I have a baby that’s not even two right now. And she already yells that, you know, she says, Hey, Google play a song. So, it’s just playing random songs at her all day. And if I’m in the bathroom or something, I feel like I’m not in control of what she’s doing or listening to.

Participant 4

Our daughter can say, Mom, Dad, and Alexa.

Participant 2

Participant feedback highlighted that voice technology used in the home setting provided instances to engage multiple users simultaneously, such as family and friends. However, the content needed to be appropriate for diverse audiences:

I mean, in terms of the content of the episodes you had us listen to... it would be great for my husband to hear and I’m fine with my kids hearing that kind of content. I didn’t think that it was inappropriate at all.

Participant 5

One participant reported not using technology to manage her stress but suggested a case of joint media engagement:

As far as technology, no. My husband will sometimes. I don’t know what the app is called, but he uses it like a meditation app sometimes when we go to bed. He’ll play it and it’s just like breathing.…it’s like breathing techniques we’ll do just to like, relax to go to bed. I make fun of him about it sometimes, but [laugh] it is very beneficial. It’s just sometimes I’m like I don’t like doing this I just want to go to bed.

Participant 2

Although not as frequent, some have also reported using voice for group entertainment. In reference to the voice assistant led trivia:

it’s pretty much music trivia and they’ll play a snippet of a music. Yes, and it’s just fun if people are over, it’s like a pastime kind of little game that doesn’t, it’s not intimidating people the whole group can play.

Participant 2

Yes, we have at friends’ houses before we don’t do it regularly though … but we should utilize the game more often because it was fun.

Participant 3

#### Theme 7: Context and Convenience

Context played a major role in how women perceived their interactions with voice technology. Women also discussed how voice interventions could be weaved into their daily routines:

in like, like a convenient setting like if I could have it, so one of the things that our Alexa does in the morning is like a morning news update and they’ll just give us like a 5 or 10 minute update about like, what’s happening in the news something like that could happen like today in your pregnancy and or like this might be a topic if you’re interested in that would be really cool then I could just listen to it while I’m getting ready in the morning.

Participant 4

Women favored voice interactions, which were convenient. A number of women reported that they did not regularly use the voice assistant on their phones (ie, Siri) because of frustration or a lack of understanding. General sentiments suggested that voice interaction could be challenging:

I mean, it’s come a long way compared to what Siri used to be used to be even worse, but in terms of what you can do, or how you ask a question matters a lot. What exactly you say matters a lot as to what type of response you’re gonna get.

Participant 9

Outside of the home setting, no voice use was preferred over Siri. In the home setting, Alexa-enabled devices were favored over Siri-enabled smartphones:

I find that Siri doesn’t work as well for me, my husband’s much better at it. I am much more comfortable using Alexa. I find that it responds better.

Yeah, well, I mean, like we use that because everything in our house is tied into Alexa … but like if I’m somewhere else, like, I won’t use Siri on my phone.

Participant 9

I do use Alexa at home and I don’t really use Siri that much on my phone though.

Participant 4

However, the value of voice-enabled smartphones increased when participants needed to be hands-free, particularly in cars. Even if a participant reported not using voice routinely on their phone, they did in the car:

No, I don’t use Siri. I’ve found that she doesn’t really understand what I say. So, I don’t tend to use it. But I do have, like, voice recognition in my car that I use a lot.

Participant 5

Especially thinking about being able to use it in the car, you know, to be able to get in and ask the app to you know, play me episodes about, you know hygiene or whatever.

Participant 6

Finally, women stated that it would be beneficial to have voice assistants to help them communicate with their health care providers and/or receive laboratory results. However, users wanted to have control over voice technology-communicated medical information:

I think it’s If it becomes convenient, and there are less hoops to jump through to be able to communicate to healthcare providers, then heck yeah, I’ll use it.

Participant 6

If I had lab results and it came through and Alexa was like, Hey – you have new lab results, do you want to hear them? I would love that…If I’m having a dinner party, I’d probably say no not right now. You know what I mean? If there’s people over, I could see why I would want to defer it.

Participant 3

Specifically, women indicated that it was more convenient to listen to the intervention compared with providing feedback, particularly relevant with regard to user context. One participant reported the following:

I actually didn’t really like it. I didn’t really want to like give the vocal feedback when I was out and about, like in my car listening to it or like in an airport... So if somebody were to give, if this were to be an app, and someone was trying to give feedback, it could be definitely a distraction thing if they’re driving and trying to give feedback.

Participant 5

## Discussion

### Principal Findings

Maternal child health remains to be a public health priority, and practices to improve outcomes are urgently needed. As an adjunct to clinical care, digital health interventions have the potential to broaden opportunities to intervene when patients may be most receptive to support [40]. The use of voice technology in digital health is nascent, particularly among perinatal populations. Thus, the goal of this study is to address the question, “Can it work?” [27] and provide evidence to support or negate the use of voice technology in perinatal digital health interventions. To address the primary research aim and determine whether voice technology is an appropriate medium to leverage in perinatal health, feasibility is framed in key focus areas: (1) acceptability, (2) demand, (3) practicality, and (4) adaptation [36].

#### Acceptability

Acceptability was examined by how the individuals reacted to the intervention, perceived ease of use, perceived helpfulness, and intention to try podcast recommendations. At baseline, users were more familiar with common smartphone features such as text messaging, calendars, and apps compared with voice technology. However, no users required additional assistance beyond onboarding to use the intervention, nor did any users provide feedback that the voice-based app was difficult to use. Of the 239 podcasts listened to by participants, sentiment ratings suggested that the content was favorably received, particularly on the topic of breastfeeding. Exit surveys indicated that women were receptive to using voice technology as a potential platform to support health. The findings also showed that participants perceived the advantages/disadvantages of voice technology depending on the device. On smartphones, voice assistants were perceived to have quick response rates, yet users complained that the technology often did not understand them or provided unsatisfactory answers. On smart speakers/devices, voice assistants were perceived as convenient, but unnecessary and unfavorable because of costs.

#### Demand

Demand was assessed by documenting intervention activities and self-reported use of technology. As a percentage, overall response rates to intervention activities (approximately 61%) suggest a fair ability/interest in completing the study tasks. Similar to other research [28], the participants in our study were technologically capable of reporting interactions with various technologies before SMILE. However, at baseline, only 1 participant reported using a smartphone to listen to podcasts, and during exit interviews, less than half of the participants reported listening to podcasts outside of the intervention. Just over half of our sample reported using voice assistants (11/19, 57.9%), slightly higher than the Pew survey data that reported 46% of Americans reported using digital voice assistants [41]. Of those with previous voice experience, 72.7% (8/11) had ≥1 year experience with smartphone voice technology and 60% (6/10) had ≥1 year experience with voice-enabled devices in the home setting. Baseline survey data and exit interviews aligned, highlighting that hands-free, convenient activities were a strength of voice technology. However, baseline surveys highlighted case-specific uses for voice technology (eg, setting a timer, checking the weather), whereas exit interviews revealed broad uses for voice technology (eg, games, communication to more than one person, answering questions). Across all sources of data collected, inability for voice technology to recognize what the user was saying was a primary reason for nonuse.

#### Practical

Participants did not find the podcasts *practical* when they were asked to refer to other podcasts for the background. Similarly, participants did not find the podcasts practical when content was not perceived as relevant to them. Participants desired content that was perceived as sensible, objective, and poised in a manner that tempered prior knowledge with new information. These findings support the use of cognitive load theory as an intervention guide, emphasizing the importance of balancing processing capacity relative to cognitive load. Regarding the practicality of intervention delivery, the voice was highly sensitive to the user context. Several participants discussed instances when intervention activities were either facilitated or constrained (ie, listening/speaking in public vs private). For instance, intervention delivery was favorably perceived during multitasking situations when users desired to be hands-free (eg, driving). Conversely, intervention activities were negatively perceived when in public settings or when with friends or colleagues outside of the woman’s immediate perinatal social support system (ie, friend vs spouse). To build upon these strengths and limitations, intervention activities should be available on demand and able to satisfy user needs at the moment. For example, the podcast duration should be able to accommodate activities such as driving, both short (10-15 minutes) and long commutes (3-4 hours).

#### Adaptations

As such, a number of *adaptations* are recommended for voice interventions to best align with user situations. Our data suggest that voice intervention activities that deliver personal or sensitive information or require quick response/feedback may be better suited for delivery on smartphones. Conversely, voice intervention activities that are more general may be better suited for delivery via smart speakers, which may also be an opportunity to engage other members of the family or social support. Although participants did not directly discuss using SMILE with others, many did see an opportunity to engage spouses and extended family members (eg, other children/siblings). In joint media engagement, more than 1 user jointly may engage with a technology and, consequently, support prescribed activities [42], which is a potential strength for voice technology. Depending on delivery and users, voice technology has the ability to support stealth health promotion, wherein efforts are perceived as an activity spent with family or friends, and the target of the intervention (eg, stress management) is a side effect but not the primary motivator of participation [43,44].

### Voice Interaction and Podcast Intervention Use

Similar to attrition found among digital health interventions [45], SMILE data suggest user attrition over time. As a prototype, SMILE was rapidly designed for this population to gauge user responses to voice technology in terms of listener preferences for duration, timing, and interaction. The sequence, number, and duration of SMILE content were fixed. After the 14th podcast, the episode duration was slightly longer (ie, further away from 5 minutes, closer to 6-8 minutes in duration). As the intervention was delivered via smartphone, users could see the title of the podcast and duration, which could bias them to listen or not. User preferences for learning may also have contributed to attrition. Some women reported personal preferences for learning (eg, *like to see things versus hearing*, *go at my own pace*), which may have been in contrast to how the intervention was designed. Given the documented need for perinatal support and value placed on digital health tools by perinatal women, particularly those tools that are multifunctional [17,28,30,46], we believe there is value in pursuing both voice and visual interventions in perinatal health. Specifically, our findings align with other research [26,47], suggesting a need to address the inefficiencies of voice navigation (eg, pace, duration, ability to choose from a list of options).

Podcasts were purposefully selected based on recommendations from the literature; therefore, our findings reaffirm previous literature and the importance of topics such as breastfeeding, infant sleep, parenting skills, and pregnancy self-care. Similarly, we also found that women prefer tailored content, which is relevant to their needs, practical, unbiased/objective, and available on demand [17,28]. However, we discovered that the potential benefit of delivering perinatal health information via voice is the ability of women to digest perinatal health content while multitasking. Thus, the intervention could be woven into daily life. However, voice interventions are highly sensitive to context. As such, design and interactivity must be agile in the user context. For example, a number of women reported listening to podcasts while driving. Voice interventions must be careful not to place individuals in harm during use (eg, distracted driving). Similarly, a number of women discussed/envisioned listening to podcasts during *3 am feedings*. Thus, postpartum voice interventions, in particular, would benefit from adaptive volume control, such as Amazon’s Alexa whisper mode.

Digital tools to support management of daily activities were common among our sample, specifically calendar use. Maternal-infant interventions deployed/accessed through voice-enabled devices in a shared setting (eg, home, car) have the potential to reach beyond the woman alone, to include social support members (eg, spouses, siblings). Perinatal interventions that aim to engage users may want to leverage such information, as research has shown that the value of something is increased if the activity can serve more than one need [44,48]. Qualitative findings from this pilot highlight opportunities to expand perinatal health promotion efforts beyond individual women to include spouses/partners using voice technology. Irrefutably, evidence shows that *pregnant women need the support of caring family members, friends, and health professionals* [49]. Research and interventions are required to provide partners of pregnant women with evidence-based information and support whole families during the perinatal period [50]. Evidence shows that men who attend antenatal care express concerns about being excluded and left feeling disappointed [30,51]. Programs that support new fathers need to help form realistic expectations, provide information ahead of time, and provide information about the possible changes in their conjugal relationship and how to develop related coping strategies. Our findings suggest that voice interventions are strengthened by individual user profiles. Voice research also suggests that users tend to explore less and choose higher-ranked items, which could be a potential limitation of individualized voice content unless options are personalized, yet diverse to expose users to broad options [26]. In exit interviews, Alexa was the most commonly mentioned smart speaker. When the study was conducted, Alexa did not support individual user profiles. However, both Google Assistant and Alexa now support individual users through voice recognition technology.

### Privacy and Security

Privacy of voice technology is complex and serves as an indicator of voice intervention acceptability. The findings reflected participant concerns over health data privacy and patient control over information sharing (ie, sharing data when and with whom). However, recent findings from a systematic review found that few voice assistant research studies reported privacy or security concerns associated with voice assistants and no studies refer to proprietary challenges that can arise when using commercial devices [25]. Therefore, we strongly advocate for transparent research and reporting when using voice, raising standards accountable to the scientific community and the participants they serve. Such methods may include lay language to explain what data are being captured, how data are shared and their intended use, potential for security breaches, and options for participants to participate fully or partially (eg, delete partial transcripts).

Although some women were enthusiastic about using voice to receive and share medical data with their health care provider, at this time, there is not enough evidence to support the safe and effective use of voice between patients and providers. Reliance on conversational assistants for actionable medical information represents a safety risk for patients, and in some instances, may pose harm [52,53]. Further research is necessary to forge confidence in voice technology and explore methods to mitigate safety risks. Developers and health technology experts should explore opportunities to broaden voice technology use; however, transparency about partnerships and data use is ethically prudent as device capabilities expand [52,54].

### Contributions to the Literature and Implications for Future Research

Findings from this feasibility study suggest a role for voice technology in maternal-infant health efforts; however, the size of the role has yet to be determined. A systematic review of voice assistant technology used in behavioral health research found that, from a limited number of studies, voice interventions were in the early stages of development with limited efficacy testing [24]. With the proliferation of voice-activated devices (eg, Apple Siri, Google Assistant, Amazon Alexa), there is substantial opportunity for empirically supported voice-enabled health solutions. Owing to dramatic improvements in voice recognition accuracy and intelligent conversational agents, voice assistant technology allows for true hands-free operation and conversation, increasing flexibility and efficiency.

Our findings further demonstrate the feasibility of capturing data from perinatal populations through voice. Voice technology is a novel strategy to collect data and interact with perinatal populations beyond the clinic. The benefit of using voice is that speaking/talking is naturalistic across diverse populations. Voice technology may help to reduce barriers associated with literacy (eg, spelling errors, mistyped words), support formative assessments, and engage social support beyond just the patient. In our future efforts, we plan to explore whether voice-captured data differ from other just-in-time data collection strategies (ie, do participants ramble or provide longer voice responses; how do voice data capture differ/align with text message data capture). Although improving, challenges to voice include errors in transcription (eg, tone, rates of speech), understanding various accents and medical terminology (eg, mispronunciation or misuse), and deducing user intent from context, that is, intent schemas to facilitate custom interactions with users [55]. Research in this area is critical to avoid user frustration and potential abandonment if the system does not understand or does not reflect user intent (ie, does not respond appropriately).

We believe that the proposed project is a vital first step necessary to elucidate how digital health, particularly voice activation, may be leveraged to promote positive perinatal health behaviors and reduce maternal/infant morbidity and mortality rates. This formative evaluation provides evidence to suggest that a perinatal educational support program using voice technology is acceptable among a group of pregnant women and has the potential to engage spousal and familial support. The findings will be iteratively combined with our team’s current efforts to partner with key stakeholders (ie, low-income families, community health workers, health care professionals) in the development of a perinatal digital health platform with voice-enabled capabilities. Future efforts focused on voice should explore the feasibility, usability, and effects of voice interventions delivered through different voice-enabled devices (eg, smartphones vs smart speakers).

### Limitations

SMILE represents the pilot work necessary to guide voice technology intervention use among perinatal populations. As such, the study was limited by a small, predominantly White, and married (2-parent) convenience sample and short study length. Similar to other digital health research in this population, moving forward, it will be necessary to engage women from socioeconomically disadvantaged backgrounds and rural locations to determine if intervention needs differ from this sample [17,54]. Detailed assessments of sociodemographic data and gauges of health and technology literacy were not captured in this study. In future research, lessons learned from this pilot will be used to conduct longer studies across diverse populations, to also include assessments of health literacy and technology proficiency. Despite efforts to reach postpartum women, our study only included pregnant women. Evidence suggests that reaching postpartum women is challenging; however, web-based recruitment strategies have been shown to increase interest/screening of postpartum women in health promotion research [56]. Other strategies worth exploring include recruiting women, infants, and children, health care providers, and mother-baby groups [57,58]. Another limitation was the basic functionality of the tested technology (voice-only interactions via mobile phones without multimodal and/or tailored content). However, the study and intervention were conducted to address the feasibility of using voice in perinatal populations and to provide a jumping off point for future research. As such, the effects of the app on perinatal health education and health outcomes were not measured.

### Conclusions

This study is one of the first attempts to develop and evaluate the feasibility of a voice technology app to promote positive self-management skills during the perinatal period using evidence-based podcasts. Our findings suggest that how pregnant women use digital health interventions differ not only between visual and voice but also between the type of device used. In addition, we collected feedback through voice interactions. The findings support further development and usability testing of voice technology to promote maternal-infant health outcomes. Given trajectory and market growth, the necessity for hands-free interaction with interactive voice devices will be a growing industry across all customer segments. The development of empirically supported interactive voice solutions should be a priority to address this inevitable need.

## Appendix

Multimedia Appendix 1 Self-Management Intervention–Life Essentials baseline survey.

Multimedia Appendix 2 Self-Management Intervention–Life Essentials exit interview guide.

Multimedia Appendix 3 Self-Management Intervention–Life Essentials list of podcasts grouped by category.

## Abbreviations

ACOG: American College of Obstetricians and Gynecologists
SMILE: Self-Management Intervention–Life Essentials
TAM: technology acceptance model

## References

[1] Petersen EE, Davis NL, Goodman D, Cox S, Mayes N, Johnston E, Syverson C, Seed K, Shapiro-Mendoza CK, Callaghan WM, Barfield W (2019). Vital signs: pregnancy-related deaths, United States, 2011-2015, and strategies for prevention, 13 states, 2013-2017. MMWR Morb Mortal Wkly Rep.

[2] Sawyer B, Gonzales S Kaiser Family Foundation: How does infant mortality in the U.S. compare to other countries?. Peterson-Kaiser Health System Tracker.

[3] Cohen S, Janicki-Deverts D (2012). Who's stressed? Distributions of psychological stress in the United States in probability samples from 1983, 2006, and 2009. J Appli Soc Psychol.

[4] Giurgescu C, Zenk SN, Engeland CG, Garfield L, Templin TN (2017). Racial discrimination and psychological wellbeing of pregnant women. MCN Am J Matern Child Nurs.

[5] Howell EA (2018). Reducing disparities in severe maternal morbidity and mortality. Clin Obstet Gynecol.

[6] Mohamoud YA, Kirby RS, Ehrenthal DB (2019). Poverty, urban-rural classification and term infant mortality: a population-based multilevel analysis. BMC Pregnancy Childbirth.

[7] (2019). American Academy of Pediatrics. Engaging patients and families: periodicity schedule.

[8] Screening recommendations. American Academy of Pediatrics.

[9] American College of Obstetricians and Gynecologists Redefining postpartum care task force. ACOG Postpartum Toolkit for Health Care Providers.

[10] (2018). Perinatal and infant health. Maternal & Child Health Topics.

[11] Tessema J, Jefferds ME, Cogswell M, Carlton E (2009). Motivators and barriers to prenatal supplement use among minority women in the United States. J Am Diet Assoc.

[12] (2018). Pregnancy risk assessment monitoring system - reproductive health. Centers for Disease Control and Prevention.

[13] (2015). Maternal health in the United States. Maternal Health Task Force.

[14] The American College of Obstetricians and Gynecologists (2018). Group versus conventional antenatal care for women. Women's Health Care Physicians.

[15] Cunningham SD, Lewis JB, Thomas JL, Grilo SA, Ickovics JR (2017). Expect with me: development and evaluation design for an innovative model of group prenatal care to improve perinatal outcomes. BMC Pregnancy Childbirth.

[16] van den Heuvel JF, Groenhof TK, Veerbeek JH, van Solinge WW, Lely AT, Franx A, Bekker MN (2018). eHealth as the next-generation perinatal care: an overview of the literature. J Med Internet Res.

[17] Lupton D (2016). The use and value of digital media for information about pregnancy and early motherhood: a focus group study. BMC Pregnancy Childbirth.

[18] Wellpass Text4baby.

[19] Cunningham SD, Lewis JB, Shebl FM, Boyd LM, Robinson MA, Grilo SA, Lewis SM, Pruett AL, Ickovics JR (2019). Group prenatal care reduces risk of preterm birth and low birth weight: a matched cohort study. J Womens Health (Larchmt).

[20] Butler Tobah YS, LeBlanc A, Branda ME, Inselman JW, Morris MA, Ridgeway JL, Finnie DM, Theiler R, Torbenson VE, Brodrick EM, Meylor de Mooij M, Gostout B, Famuyide A (2019). Randomized comparison of a reduced-visit prenatal care model enhanced with remote monitoring. Am J Obstet Gynecol.

[21] de Mooij MJM, Hodny RL, O'Neil DA, Gardner MR, Beaver M, Brown AT, Barry BA, Ross LM, Jasik AJ, Nesbitt KM, Sobolewski SM, Skinner SM, Chaudhry R, Brost BC, Gostout BS, Harms RW (2018). OB nest: reimagining low-risk prenatal care. Mayo Clin Proc.

[22] Gardiner PM, McCue KD, Negash LM, Cheng T, White LF, Yinusa-Nyahkoon L, Jack BW, Bickmore TW (2017). Engaging women with an embodied conversational agent to deliver mindfulness and lifestyle recommendations: a feasibility randomized control trial. Patient Educ Couns.

[23] Gardiner P, Hempstead MB, Ring L, Bickmore T, Yinusa-Nyahkoon L, Tran H, Paasche-Orlow M, Damus K, Jack B (2013). Reaching women through health information technology: the Gabby preconception care system. Am J Health Promot.

[24] Sezgin E, Militello L, Huang Y, Lin S (2019). A scoping review of patient-facing, behavioral health interventions with voice assistant technology targeting self-management and healthy lifestyle behaviors. SSRN Journal.

[25] Halili L, Liu R, Hutchinson KA, Semeniuk K, Redman LM, Adamo KB (2018). Development and pilot evaluation of a pregnancy-specific mobile health tool: a qualitative investigation of SmartMoms Canada. BMC Med Inform Decis Mak.

[26] Slomian J, Emonts P, Vigneron L, Acconcia A, Glowacz F, Reginster JY, Oumourgh M, Bruyère O (2017). Identifying maternal needs following childbirth: a qualitative study among mothers, fathers and professionals. BMC Pregnancy Childbirth.

[27] Shorey S, Ng ED (2019). Evaluation of mothers' perceptions of a technology-based supportive educational parenting program (part 2): qualitative study. J Med Internet Res.

[28] Cho D, Cosimini M, Espinoza J (2017). Podcasting in medical education: a review of the literature. Korean J Med Educ.

[29] Parga-Belinkie J, Merchant RM (2019). Voices in evidence-based newborn care: a how-to-guide on developing a parent-facing podcast. JMIR Pediatr Parent.

[30] Mike Patrick PediaCast - a pediatric podcast for parents.

[31] Davis FD (1989). Perceived usefulness, perceived ease of use, and user acceptance of information technology. MIS Quarterly.

[32] Suh H, Shahriaree N, Hekler E, Kientz J (2016). Developing and validating the user burden scale: a tool for assessing user burden in computing systems. Computer Human Interaction.

[33] Ayala GX, Elder JP (2011). Qualitative methods to ensure acceptability of behavioral and social interventions to the target population. J Public Health Dent.

[34] Braun V, Clarke V (2006). Using thematic analysis in psychology. Qual Res in Psychol.

[35] Maguire M, Delahunt B (2017). Doing a thematic analysis: a practical, step-by-step guide for learning and teaching scholars.

[36] Bowen DJ, Kreuter M, Spring B, Cofta-Woerpel L, Linnan L, Weiner D, Bakken S, Kaplan CP, Squiers L, Fabrizio C, Fernandez M (2009). How we design feasibility studies. Am J Prev Med.

[37] Usability Testing. Usability.gov.

[38] Pew Research Center (2017). Nearly half of Americans use digital voice assistants, mostly on their smartphones.

[39] Eysenbach G (2005). The law of attrition. J Med Internet Res.

[40] Yang L, Sobolev M, Tsangouri C, Estrin D (2018). Understanding user interactions with podcast recommendations delivered via voice. Proceedings of the 12th ACM Conference on Recommender Systems.

[41] Turner-McGrievy G, Kalyanaraman S, Campbell MK (2013). Delivering health information via podcast or web: media effects on psychosocial and physiological responses. Health Commun.

[42] Ickovics JR, Lewis JB, Cunningham SD, Thomas J, Magriples U (2019). Transforming prenatal care: multidisciplinary team science improves a broad range of maternal-child outcomes. Am Psychol.

[43] Takeuchi L, Stevens R (2011). The new coviewing: designing for learning through joint media engagement. Sesame workshop.

[44] Wearing JR, Nollen N, Befort C, Davis AM, Agemy CK (2014). iPhone app adherence to expert-recommended guidelines for pediatric obesity prevention. Child Obes.

[45] Militello L, Hanna N, Nigg C (2018). Leveraging family to promote digital health: findings from the retrospective pokémon go family study. JMIR Pediatrics and Parenting.

[46] Kruglanski AW, Shah JY, Fishbach A, Friedman R, Chun WY, Sleeth-Keppler D (2002). A theory of goal systems. Advances in experimental social psychology.

[47] Bickmore TW, Trinh H, Olafsson S, O'Leary TK, Asadi R, Rickles NM, Cruz R (2018). Patient and consumer safety risks when using conversational assistants for medical information: an observational study of Siri, Alexa, and Google Assistant. J Med Internet Res.

[48] Miner AS, Milstein A, Schueller S, Hegde R, Mangurian C, Linos E (2016). Smartphone-based conversational agents and responses to questions about mental health, interpersonal violence, and physical health. JAMA Intern Med.

[49] Alagha EC, Helbing RR (2019). Evaluating the quality of voice assistants' responses to consumer health questions about vaccines: an exploratory comparison of Alexa, Google Assistant and Siri. BMJ Health Care Inform.

[50] East CE, Biro MA, Fredericks S, Lau R (2019). Support during pregnancy for women at increased risk of low birthweight babies. Cochrane Database Syst Rev.

[51] Suto M, Takehara K, Yamane Y, Ota E (2017). Effects of prenatal childbirth education for partners of pregnant women on paternal postnatal mental health and couple relationship: a systematic review. J Affect Disord.

[52] Entsieh AA, Hallström IK (2016). First-time parents' prenatal needs for early parenthood preparation- a systematic review and meta-synthesis of qualitative literature. Midwifery.

[53] Baldwin S, Malone M, Sandall J, Bick D (2018). Mental health and wellbeing during the transition to fatherhood: a systematic review of first time fathers' experiences. JBI Database System Rev Implement Rep.

[54] Guendelman S, Broderick A, Mlo H, Gemmill A, Lindeman D (2017). Listening to communities: mixed-method study of the engagement of disadvantaged mothers and pregnant women with digital health technologies. J Med Internet Res.

[55] Kumah-Crystal Yaa A, Pirtle Claude J, Whyte Harrison M, Goode Edward S, Anders Shilo H, Lehmann Christoph U (2018). Electronic Health Record Interactions through Voice: A Review. Appl Clin Inform.

[56] Leach Liana S, Butterworth Peter, Poyser Carmel, Batterham Philip J, Farrer Louise M (2017). Online Recruitment: Feasibility, Cost, and Representativeness in a Study of Postpartum Women. J Med Internet Res.

[57] Haste Anna, Adamson Ashley J, McColl Elaine, Araujo-Soares Vera, Bell Ruth (2018). Problems recruiting and retaining postnatal women to a pilot randomised controlled trial of a web-delivered weight loss intervention. BMC Res Notes.

[58] Silfee Valerie J, Lopez-Cepero Andrea, Lemon Stephenie C, Estabrook Barbara, Nguyen Oanh, Wang Monica L, Rosal Milagros C (2018). Adapting a Behavioral Weight Loss Intervention for Delivery via Facebook: A Pilot Series Among Low-Income Postpartum Women. JMIR Form Res.

