# Supplementary material for: Delivering Perinatal Health Information via a Voice Interactive App (SMILE): Mixed Methods Feasibility Study
Source: JMIR Form Res. 2021 Mar 1;5(3):e18240. doi: 10.2196/18240 (PMC7961402; doi:10.2196/18240)
Supplement: Multimedia Appendix 1 [file formative_v5i3e18240_app1.pdf]

SMILE introduction survey

Please complete the survey below.

Thank you!

Name & Surname ______________________

Contact information (email or phone) ______________________

App ID (please see image below showing how to access APP ID) ______________________
To complete this part, you need to install the app from the App store using the link provided in study introduction packet or link below. Once installed, Please enter 43205 access code to initiate the app.

https://itunes.apple.com/us/app/smile-by-nationwide-childrens/id1453094747

Please see the image for accessing your SMILE app ID

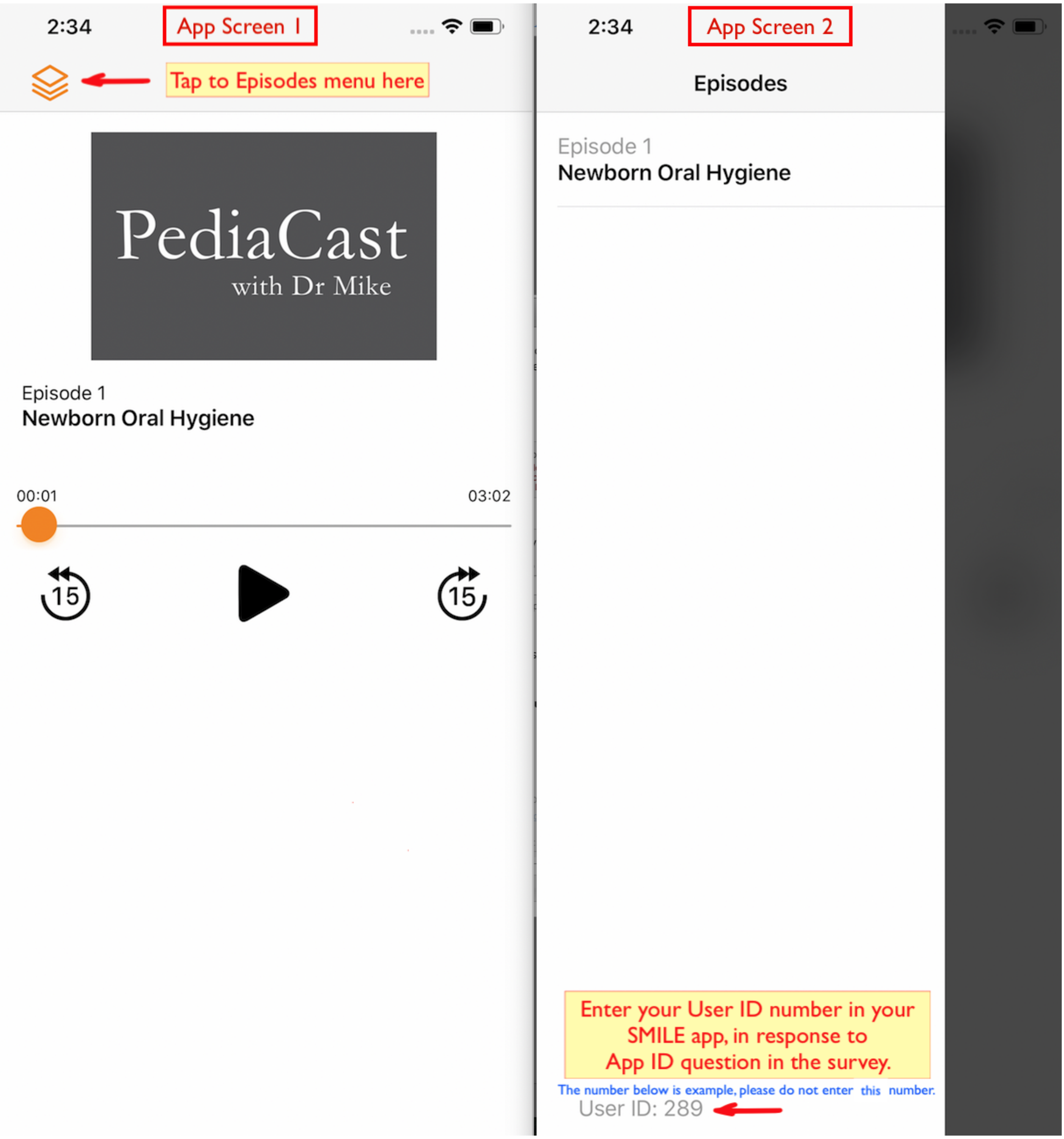

What is your age?

- ◯ 18-24 years old
- ◯ 25-34 years old
- ◯ 35-44 years old
- ◯ 45-or older
- ◯ Prefer not to answer

How do you define your current family?	◯ Single mother household<br>◯ Two parent household<br>◯ Single mother plus grandparent living in household<br>◯ Single mother plus extended family or friend living in household<br>◯ Other<br>◯ Prefer not to answer
How do you identify yourself (race/ethnicity)?	◯ White<br>◯ Latino, or Spanish origin<br>◯ African American<br>◯ American Indian or Alaska Native<br>◯ Asian/Pacific Islander<br>◯ Other<br>◯ Prefer not to answer
Are you currently pregnant?	◯ Yes<br>◯ No
How many children (18 years or younger) live at home/with you?	◯ 0 (I am pregnant to my first child)<br>◯ 1<br>◯ 2<br>◯ 3<br>◯ 4<br>◯ 5+<br>◯ Prefer not to answer
Which city or location do you live in? Please enter the zip code.	__________________________________

Voice interaction questions

Do you interact with your smartphone with voice (e.g. using Google assistant - "Hey Google!", Siri or Alexa app)?	◯ Yes<br>◯ No
What are your top 3 voice interactions with your smartphone?	____
For how long have you been using voice interaction with your phone?	◯ 1-3 months<br>◯ 3-12 months<br>◯ 1-3 years<br>◯ More than 3 years
What is the main reason to prefer or not prefer to use voice interaction with your phone?	____
Do you use voice-interactive devices / smart speakers (e.g. Amazon Alexa, Google Home, Apple HomePod)?	◯ Yes<br>◯ No
What are your top 3 voice interactions with your smart speaker?	____

For how long have you been using voice interaction with your smart speaker?	◯ 1-3 months<br>◯ 3-12 months<br>◯ 1-3 years<br>◯ More than 3 years
What is the main reason to prefer or not prefer to use voice interaction with a smart speaker?	__________________________________

Mobile use questions

What are your frequently used smartphone applications (Top 3) in everyday life?	__________________________________
What are your frequently used smartphone applications (Top 3) related to your baby and/or pregnancy?	__________________________________
What is your primary source of information regarding pregnancy? (E.g. web, mobile app, or calling friend, nurse or doctor)	__________________________________

Self management questions

Do you use any technology for your self-management?	◯ Yes<br>◯ No
What do you use? please briefly explain.	____________________________
For how long have you been using technology in self-management?	◯ 1-3 months<br>◯ 3-12 months<br>◯ 1-3 years<br>◯ More than 3 years
Do you usually practice managing your stress? (such as meditating, mindfulness exercises)	◯ Yes<br>◯ No
What do you do specifically? please briefly explain.	____________________________
For how long have you been using technology in stress-management?	◯ 1-3 months<br>◯ 3-12 months<br>◯ 1-3 years<br>◯ More than 3 years
What would be your ideal self management tool considering current technologies existing?	__________________________________
