# Supplementary material for: Delivering Perinatal Health Information via a Voice Interactive App (SMILE): Mixed Methods Feasibility Study
Source: JMIR Form Res. 2021 Mar 1;5(3):e18240. doi: 10.2196/18240 (PMC7961402; doi:10.2196/18240)
Supplement: Multimedia Appendix 2 [file formative_v5i3e18240_app2.docx]

## **Multimedia Appendix 2.** Self-Management Intervention–Life Essentials exit interview guide.

## **Introduction**

Thank you for joining us today for this focus group interview. We will talk about voice interactive technologies and your experience. And discuss how we can utilize this technology in self-management during pregnancy.

SMILE is to let you experience voice interaction, but the following questions are not focused on your experience with SMILE but understanding your thoughts on voice interaction.

## **Transitioning**

Could you summarize a regular day of your life? For example, any routines or things that you typically do. Sometimes your routines differ during the week versus on a weekend. Can you tell us about your typical day M-F. What about on the weekends? Sat-Sun?

What technologies do you typically use? (if participant unable to readily provide answer, give examples such as phones, tv, video game)

**Follow-up questions based on the survey**

Elaborating survey responses:

- What are the main motivations to use voice interaction with your smartphone?
- What are the main motivations to use voice interactive devices?
- What are the main motivations to use the apps you mentioned in the survey?

Can you think of anything in particular that might cause stress in your life or is causing you stress?

What do you typically do to handle your stress?

Have you ever used any kind of technology to help you manage stress?  Why or why not?

## **Voice interaction - focused questions**

## **Adoption**

**TAM**

**Perceived Ease of Use**

Do you think voice-interaction is an easy to use method compared the other apps you use? How so?

What do you think about the content? did you like listening instead of reading?

What troubles did you have with voice feedback on SMILE?

**Perceived Usefulness**
Do you think voice interactive apps/skills could be useful in educating you about how to take care of yourself and your baby- like SMILE podcasts?

Do you think voice interactive apps/skills could be useful in teaching you skills about how to take care of yourself and your baby -like having exercise or training sessions?

Do you think voice-interaction is useful way to communicate health care? Can you think of any pros or cons?

**Behavioral Intention:**

Would you use SMILE or similar voice-interactive apps in the future to help you take care of yourself and your baby? Why or why not?

Do you think listening the content would help more than reading ? -- would you like listening podcasts in general?

Do you have recommendations for content or features that should be in an app for pregnant women or new mothers?

Would you like the app/skill more if it was personalized or tailored to your interests? If so, any suggestions?

**Burden scale**

**Difficulty of use**

How long do you think the audio content should be? For example, were the podcasts too short or too long?

**Physical**

What do you think about physical effort to use voice interactive app or skill vs mobile app? Would you prefer to only talk to the app or would you like features where you can scroll through content also?

**Time and social**

Would voice interactive apps have any effect on your daily relationships or social life? (positive or negative)

**Mental and emotional**

Do you feel like you need to remember more than any other apps while using a voice interactive app or skill? Such as, wake word, command, etc.

Do voice-interactive app or skills make you feel emotionally any different? Feeling better for knowing the content you received in the given time period or feeling worse for spending time with app or receiving these information?

**Privacy**

Did you have any concerns about your privacy while using voice-interactive apps or skills?

What are your thoughts about the system recording your voice feedback?

**Financial**

Do you pay for any health information services? Would you consider paying for such services about perinatal care and baby care if you receive periodically relevant contents over a voice activated device? --- did you ever pay for apps? If it is not private for you, could you tell me what apps did you pay to purchase?

### **Future use**

How would you envision the future use of voice interactive technologies for pregnant women in the future?

How would you be use such a technology with your spouse, friends and family members?

How would you use such a technology with your care providers? What information would like them using this technology?

## **Closing**

Of all the things we discussed, what to you is the most important?

Have we missed anything? Anything you would like to add?
