# Supplementary material for: Delivering Perinatal Health Information via a Voice Interactive App (SMILE): Mixed Methods Feasibility Study
Source: JMIR Form Res. 2021 Mar 1;5(3):e18240. doi: 10.2196/18240 (PMC7961402; doi:10.2196/18240)

**Multimedia Appendix 3.** Self-Management Intervention–Life Essentials list of podcasts grouped by category.


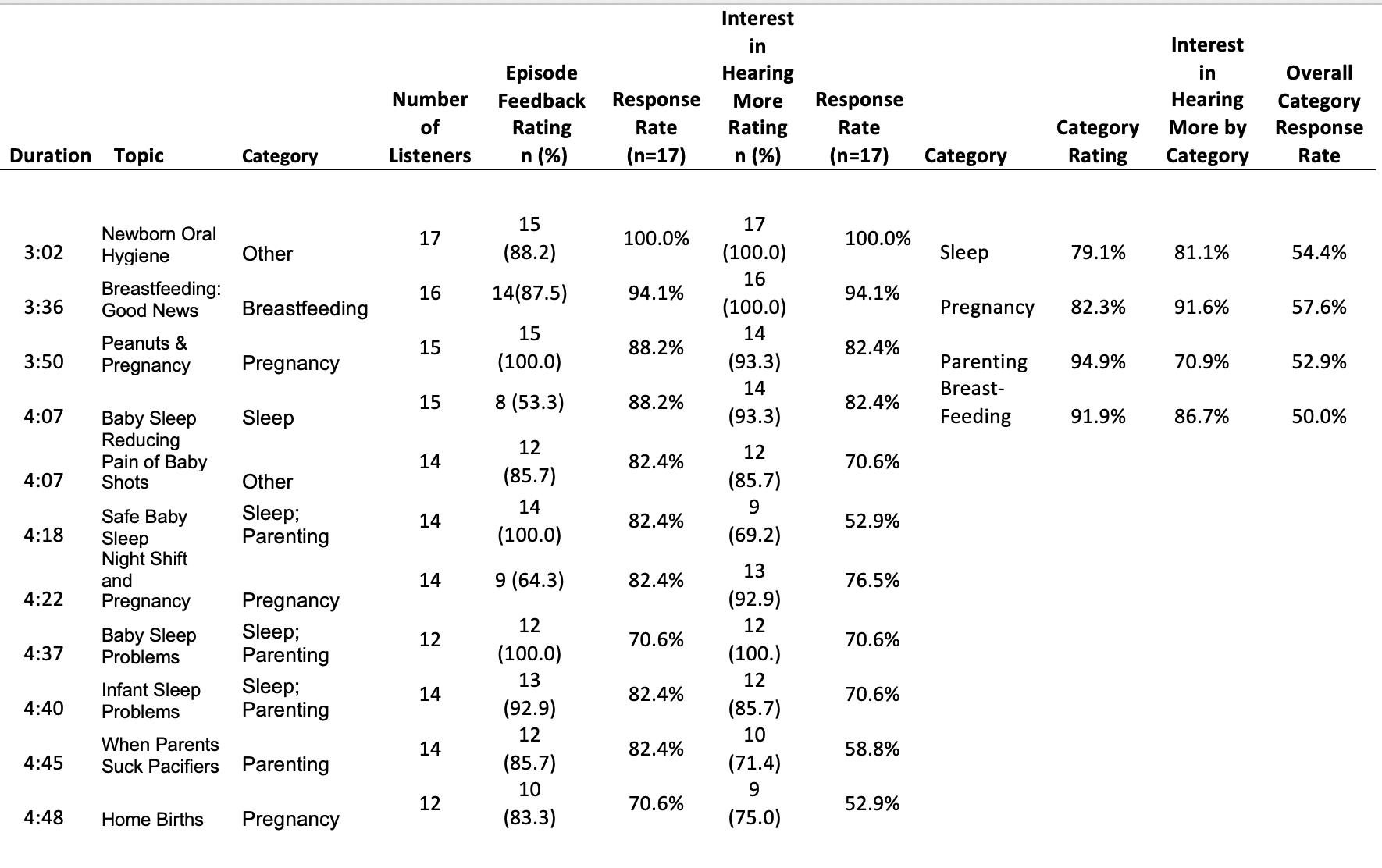


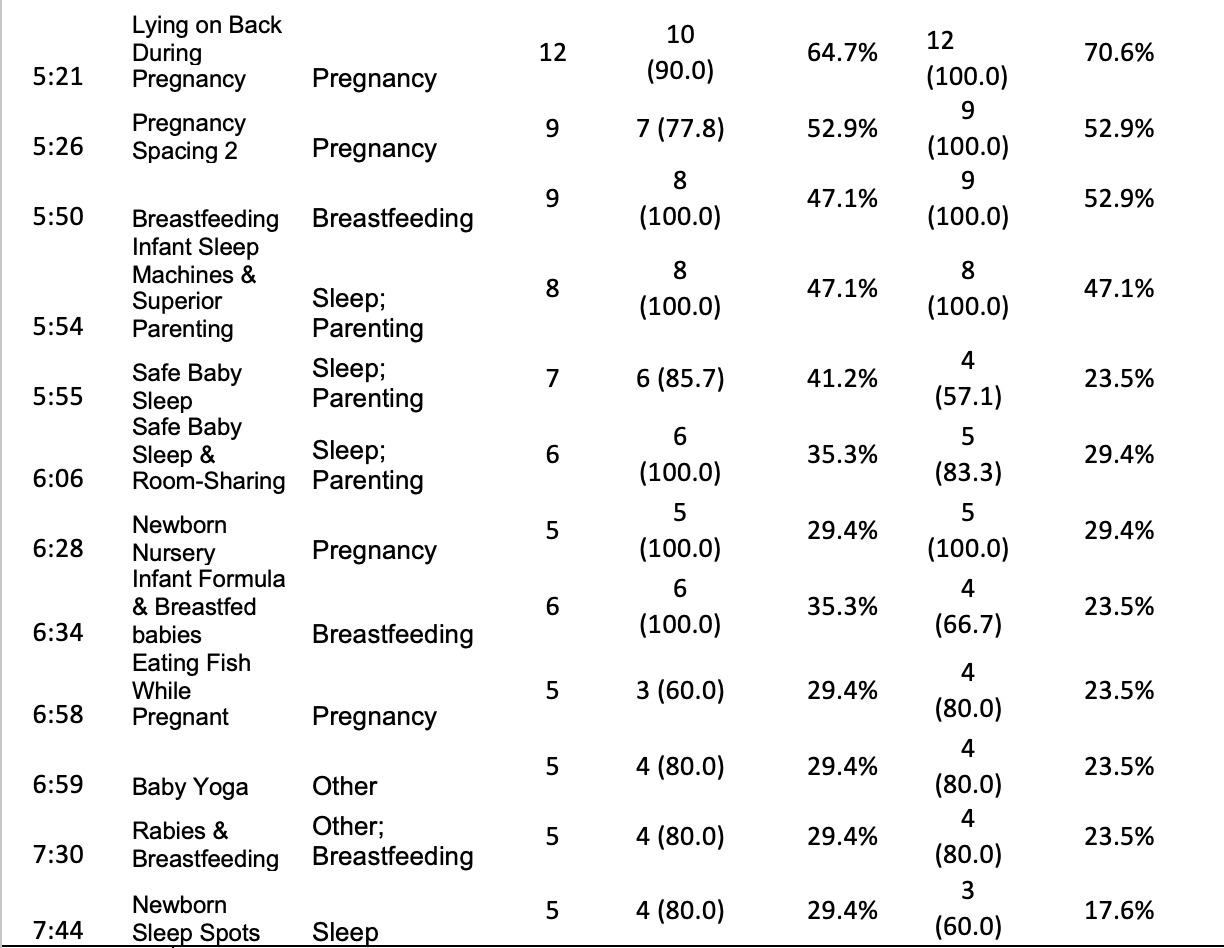

Supplement: Multimedia Appendix 3 [file formative_v5i3e18240_app3.docx]
